# ﻿*Solanumscalarium* (Solanaceae), a newly-described dioecious bush tomato from Judbarra/Gregory National Park, Northern Territory, Australia

**DOI:** 10.3897/phytokeys.216.85972

**Published:** 2022-12-20

**Authors:** Tanisha M. Williams, Jonathan Hayes, Angela J. McDonnell, Jason T. Cantley, Peter Jobson, Christopher T. Martine

**Affiliations:** 1 Department of Biology, Bucknell University, 1 Dent Drive, Lewisburg, PA, USA Bucknell University Lewisburg United States of America; 2 Negaunee Institute for Plant Conservation Science and Action, Chicago Botanic Garden, 1000 Lake Cook Rd, Glencoe, IL 60022, USA Negaunee Institute for Plant Conservation Science and Action, Chicago Botanic Garden Glencoe United States of America; 3 Department of Biology, San Francisco State University, 1600 Holloway Avenue, San Francisco, CA 96132, USA San Francisco State University San Francisco United States of America; 4 Northern Territory Herbarium, Alice Springs, Department of Environment, Parks and Water Security, Alice Springs, Northern Territory, 0870, Australia Northern Territory Herbarium Alice Springs Australia

**Keywords:** Australia, dioecy, inaperturate pollen, Judburra/Gregory National Park, new species, Northern Territory, Solanaceae, *Solanumdioicum*

## Abstract

A new species of functionally dioecious bush tomato of SolanumsubgenusLeptostemonum is described. *Solanumscalarium* Martine & T.M.Williams, **sp. nov.**, is a member of the taxonomically challenging “Kimberley dioecious clade” in Australia and differs from other species in the group in its spreading decumbent habit and conspicuously prickly male floral rachis. The species is so far known from one site in Judbarra/Gregory National Park in the Northern Territory. Ex situ crosses and confirmation of inaperturate pollen grains produced in morphologically cosexual flowers indicate that these flowers are functionally female and the species is functionally dioecious. The scientific name reflects the ladder-like appearance of the inflorescence rachis armature of male individuals, the stone staircase that provides access to the type locality at the Escarpment Lookout Walk, and the importance of maintaining equitable and safe access to outdoor spaces. The common name Garrarnawun Bush Tomato is proposed in recognition of the lookout point at this site, a traditional meeting place of the Wardaman and Nungali-Ngaliwurru peoples whose lands overlap in this area.

## ﻿Introduction

*Solanum* L. is the most species-rich genus in the family Solanaceae and among the largest in the angiosperms, with ca. 1400 accepted species distributed on every continent except Antarctica (Gagnon et al. 2022). Much of the richness of the genus is concentrated in circum-Amazonian tropical South America, but other hotspots include Africa and Australia (Symon 1981; Särkinen et al. 2013; Vorontsova et al. 2013; Gagnon et al. 2022). The genus is often recognized by its pentamerous flowers with fused sepals and petals, five stamens, 2-chambered superior ovary, poricidal anthers, and, in many species, branched hairs and/or prickles (Knapp 2013). Solanums exhibit great diversity both in vegetative and reproductive traits (especially in floral and fruit traits), ecology, and reproductive biology (see Hilgenhof et al. in review).

**Figure 1.**
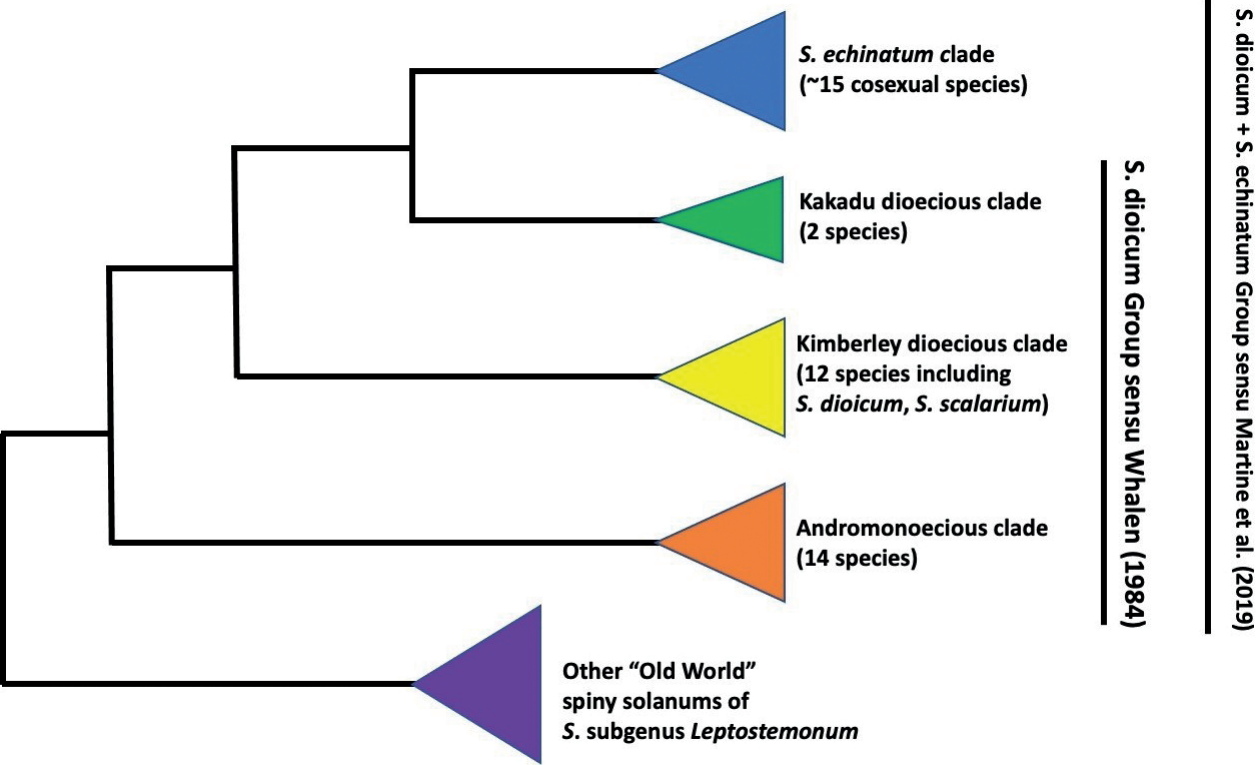
Representational phylogeny showing hypothesized relationships among the clades included in the “*S.dioicum* + *S.echinatum* Group” sensu Martine et al. (2019), based on that study plus forthcoming work by McDonnell and Martine (in prep). *Solanumscalarium* sp. nov. is one of twelve described species in the “Kimberley dioecious clade” sensu Martine et al. (2006), a clade of functionally dioecious species (and phrase-named morphological variants of *S.dioicum*) sometimes also referred to as the “Dioicum Complex”.

Despite decades of research on phylogenetic relationships within *Solanum*, there is still a great deal of work to be done to fully understand the evolutionary history of this hyper diverse group. This challenge arises, in part, due to the large number of species already described within the genus coupled with a large number of species still being described. In the past decade alone, there have been more than 100 newly described *Solanum* species (see McDonnell et al. 2019). One hotspot for new descriptions over that period has been northern Australia (e.g., Brennan et al. 2006; Bean and Albrecht 2008; Barrett 2013; Martine et al. 2013, 2016a, c; Bean 2016; Lacey et al. 2017; McDonnell et al. 2019). This area is home to a clade of ca. 45 currently described species of “spiny solanums” (i.e., SolanumsubgenusLeptostemonum Bitter, the Leptostemonum Clade) belonging to the *S.dioicum* + *S.echinatum* Group sensu Martine et al. (2019) (see Fig. 1 for breakdown of clade names related to this group). Key morphological characteristics of Leptostemonum Clade include the presence of stellate pubescence, stems and leaves with prickles, and attenuate anthers (Whalen 1984).

**Figure 2.**
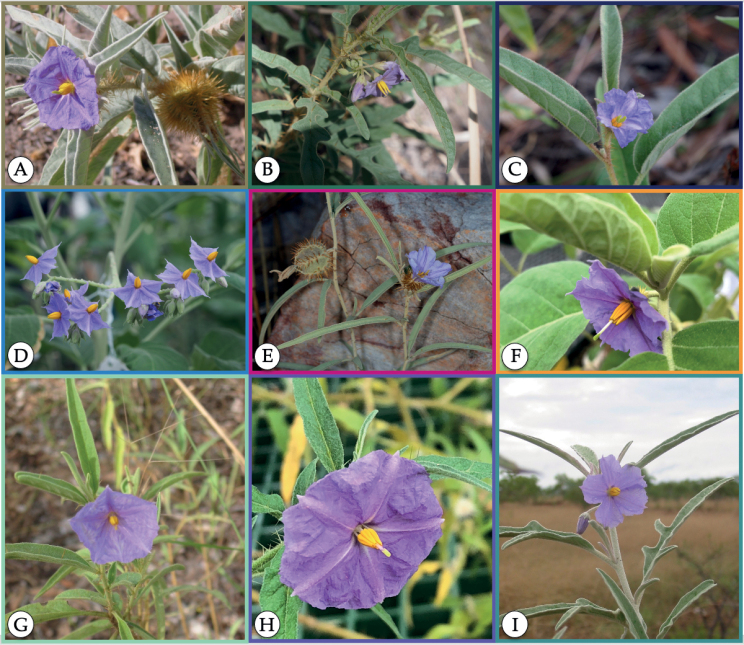
Functionally dioecious species of the “*S.dioicum* + *S.echinatum* Group.” **A***S.ossicruentum* Martine & J.Cantley **B***S.carduiforme* F.Muell. **C***S.dioicum* W.Fitzg. **D***S.asymmetriphyllum* Specht **E***S.cowiei* Martine **F***S.sejunctum* K.Brennan, C.Martine & Symon **G***S.petraeum* Symon **H***S.leopoldense* Symon and **I***S.tudununggae* Symon. *Solanumcataphractum* Cunn. ex Benth., *S.cunninghamii* Benth., *S.scalarium* Martine & T.M.Williams, *S.vansittartense* C.Gardner, and *S.zoeae* R.L.Barrett not pictured here. Colors are associated with the distribution map shown in Fig. 3. (Photos by C.T. Martine except for E by K. Brennan.).

Phylogenetic work has uncovered two Australian clades (Fig. 1) of functionally dioecious *Solanum* species: the “Kakadu dioecious clade” (two species [plus one forthcoming] of the upper Northern Territory) and the “Kimberley dioecious clade” (12 species occurring from the Kimberley Plateau of Western Australia to far northwestern Queensland (Bean 2004; Martine et al. 2006; Martine et al. 2009; Martine et al. 2019; McDonnell and Martine 2020; Figs 1, 2). The “Kimberley dioecious clade” is a well-supported clade of usually clonal shrub taxa that have proven to be taxonomically challenging, ostensibly due to a complex (or at least quite recent) evolutionary history (Symon 1981; Martine et al. 2006, 2009). Species boundaries are often blurred by overlapping or intermediate morphological traits, making it sometimes difficult to distinguish taxa in the field (Symon 1981; Martine et al. 2016c); the employment of molecular phylogenetic data to address this challenge has so far resulted in poorly-resolved intraclade relationships (see Gagnon et al. 2022).

Judburra/Gregory National Park, where the new species described here is found, is floristically diverse as a result of its sandstones and limestones that have been weathered to produce deep gorges and escarpments that sustain a diversity of habitats (Australian Government Department of the Environment and Energy 2015). In recent decades the Northern Territory government has funded a number of monitoring and vegetation surveys throughout the territory. These have resulted in a series of papers describing new endemic species to the region (e.g., Craven 1998; Walsh and Albrecht 1998; Jobson 2014; Martine et al. 2016a), and others recognized as phrase-name taxa — i.e., potentially-new species recognized as distinct variants by regional field botanists (e.g., Cowie et al. 2017). On a recent collecting trip to the region as part of phylogenetic and biogeographical studies on the flora of the Australian Monsoon Tropics (AMT), a *Solanum* population was recognized as a possibly new species by CTM because of its spreading decumbent habit and its unusual staminate inflorescence axis armed with relatively stout, spreading, straight prickles (Fig. 6) and is described here as *Solanumscalarium* Martine & T.M.Williams, sp. nov. This taxon is one of the many “Kimberley dioecious clade” variants found throughout north and northwestern Australia (Symon 1981; Purdie et al. 1982; Barrett 2013).

## ﻿Methods

A single fertile voucher specimen collected from the type locality included mature fruits. Once back at Bucknell University, seeds from those fruits were removed and germinated in order to build a living collection of greenhouse plants to better assess the morphology of this putative new species. Seeds were germinated following a 24-hour soak in 1,000-ppm gibberellic acid and sown in a controlled growth chamber environment following Martine et al. (2016a). Mature plants were cultivated in an IPM-managed greenhouse following Hayes et al. (2019). Observations of the taxon by JTC, PJ, CTM, and AJM in Judbarra/Gregory National Park (NT) are combined here with measurements of characters by JH and TMW from plants grown in cultivation (Figs 4–6). The morphological description is based mostly on those cultivated individuals because of limited herbarium material of the species; data in the Australasian Virtual Herbarium and physical examination of “*S.dioicum*” holdings at the Northern Territory Herbaria (DNA, NT) suggest the species is not represented in collections beyond the type collections cited below. A map (Fig. 2) comparing the distributions of dioecious *Solanum* species in Australia was generated using records from the Australasian Virtual Herbarium.

## ﻿Taxonomic treatment

### ﻿ Solanum scalarium

Taxon classificationPlantaeSolanalesSolanaceae

Martine & T.M.Williams sp. nov.

20A0783F-AF3A-5D4C-967C-95514A3698D7

urn:lsid:ipni.org:names:77310466-1

Figs 4 , 5 , 6 , 7

#### Diagnosis.

This species is distinguished from *Solanumdioicum* W.Fitzg. (as currently delineated) and other Australian functionally dioecious *Solanum* species of the “Kimberley dioecious clade” by the combination of a spreading decumbent habit and the staminate inflorescence axis armed with relatively stout, spreading, straight prickles.

#### Type.

Australia. Northern Territory: Victoria River Valley, Judbarra National Park, off Victoria Highway (Highway 1), NW of Victoria River Roadhouse, Escarpment Walk, just off track above Garrarnawun Lookout on flat area between there and peak of outcrop, 15.61054°S, 131.11571°E, elev. 167 m, 2 June 2018 (fr), *C. T. Martine, J. Cantley, A. McDonnell & P. Jobson 4748* (holotype: DNA).

#### Description.

Perennial spreading decumbent pale green shrub up to 30 cm tall. Main stem single, 4–12 cm tall, woody (not corky) branching 2–4 times with thickest lateral stems ca. 2–4 cm in diameter; younger stems yellow-green to tan-green in color and older woody stems eventually becoming dark tan or gray. Internodes 12–40 cm long in male plants, 30–46 cm long in functionally female plants. ***Stems*** with short, dense indumentum of porrect-stellate trichomes 0.5–1.3 mm, these mostly short stalked (occasionally on longer stalks up to 1 mm) with central midpoint ca. 0.2 mm. Prickles abundant and dense (8–10 per cm of internode), 1–8 mm long, straight, fine, widened at base, somewhat sharp. ***Leaves*** simple; blades 5–9 cm long, 1–3 cm wide, alternate, lanceolate; unarmed or with 1–3 straight prickles along adaxial midvein, soft yellow green above, slightly lighter beneath, both sides densely stellate-hairy, trichomes mostly short stalked, porrect-stellate with short central ray; apex acuminate; margins entire, sometimes ciliate; base oblique and tapering; petiole 0.5–14 mm long; ***Male inflorescence*** a scorpioid cyme 9–24 mm long with up to ca. 50 flowers (typically 1–4 flowers open at a time with previous blooms abscised); rachis densely stellate-pubescent, armed with straight prickles 5–7 mm, ca. 1 mm in diameter at the base, each subtending a flower; pedicel 3–7 mm long, sparsely armed with small prickles. ***Male flowers*** 5-merous; calyx with the tube 6–7 mm long, campanulate, armed with weak prickles ca. 2 mm long, the lobes 3–4 mm long, tipped with a linear acumen; corolla 16–27.4 mm in diameter, rotate to rotate-campanulate, pale violet; stamens equal; filaments 1–2 mm long; anthers ca. 4 mm long, tightly connivent, oblong-lanceolate to somewhat tapered, poricidal at the tips; ovary vestigial, non-functional. ***Female inflorescence*** of a solitary, morphologically cosexual flower (functionally female and producing inaperturate pollen); pedicel 7–8 mm long, sparsely armed with small prickles ca. 2 mm. ***Female flowers*** 5-merous; on; calyx with the tube campanulate, densely stellate-pubescent and armed, the prickles 5–6 mm long, straight, the lobes 5–11 mm long, unequal, long-triangular with a linear acumen, prickly; corolla 36–46 mm in diameter, rotate to rotate-campanulate, violet to pale violet; stamens equal, like those of the male flowers; filaments 1–2 mm long; anthers ca. 4 mm long, slightly spreading, poricidal at the tips; ovary ca. 5 mm in diameter at anthesis, glabrous; style ca. 5 mm long (including stigmatic surfaces), straight; stigma yellow, bifid, the lobes 1.5–2 mm long. ***Fruit*** a berry, 20–25 mm diameter, globose; immature fruit green, fleshy; mature fruit light green, drying to yellow-orange or tan, becoming leathery-reticulate and bony hard and loosely retained and partly-enclosed in calyx (75% enclosed when developing; mature, hardened fruit less than 25% enclosed), apparently detaching from calyx once hard and dry. Fruiting calyx lobes 2.1–2.8 cm long, long acuminate, tapered to a long fine tip, accrescent, slightly sticky and adherent to fruit when immature, readily separating from fruit as the berry matures, hardens, and shrinks from drying, densely armed with sharp prickles ca. 6 mm long. ***Seeds*** up to 420–586 per fruit in cultivation (two wild-collected fruits were *N* = 96 and *N* = 162), 1.1–1.5 mm in diameter, reniform dark brown to black, conspicuously and minutely reticulate.

#### Distribution and ecology.

**Figure 3.**
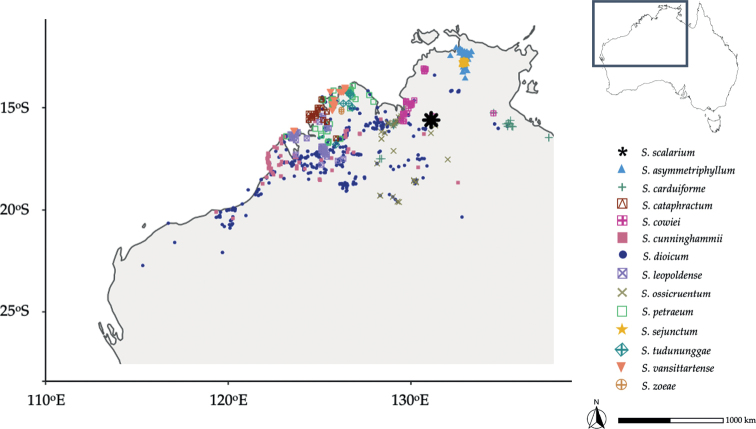
Geographic distribution of *Solanumscalarium* (black star) and other Australian dioecious *Solanum* species (source: The Australasian Virtual Herbarium).

**Figure 4.**
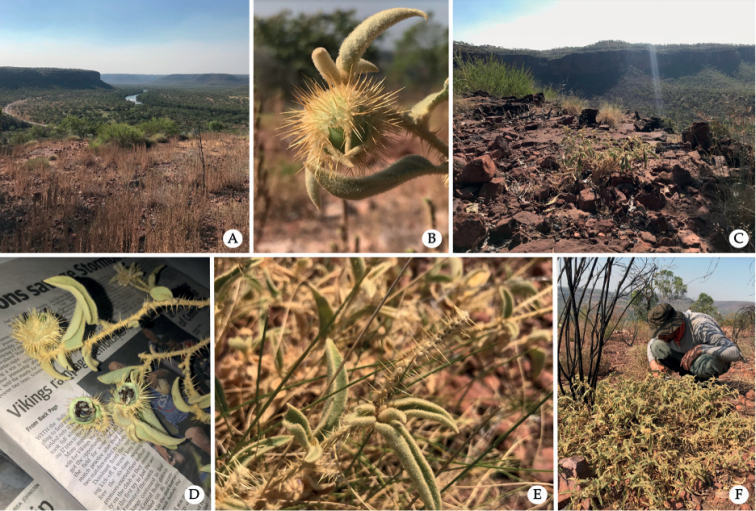
*Solanumscalarium* in the field **A, C, F** type locality and habitat, Escarpment Walk, Judbarra National Park, Northern Territory **B–D** immature green fruits enclosed in prickly calyx and **E** prickly male rachis after male flowers have dropped off. (Photos by A.J. McDonnell.).

*Solanumscalarium* is presently known from a single population (Fig. 3) of perhaps 50–100 individuals found within Judbarra/Gregory National Park. The species here occurs on skeletal pink soil, exposed sandstone pavement and dissected rock high above the Victoria River Valley (Fig. 4). The associated vegetation at this site is a low open woodland dominated by *Corymbiaterminalis* (F.Muell.) K.D.Hill & L.A.S.Johnson (Myrtaceae) and *Eucalyptusminiata* (F.Muell.) A.Cunn. ex Schauer (Myrtaceae), with a sparse low mid story of *Oweniavernicosa* (F.Muell.) (Meliaceae), *Calytrixexstipulata* DC. (Myrtaceae), *Xanthostemonparadoxus* (F.Muell.) W.J.Hooker (Myrtaceae), *Hibbertia* spp. (Dilleniaceae), *Corchorus* spp. (Malvaceae), *Sennaoligoclada* (F.Muell.) Randell (Fabaceae), *Acacia* sp. nov. (Fabaceae); the sparse ground layer is dominated by *Cyperuscunninghamii* (C.B.Clarke) C.A. Gardner (Cyperaceae) and *Triodiapungens* R.Br. (Poaceae). Although *S.scalarium* was not conspicuous on a visit by PJ and JTC in 2017, it appeared in 2018 to have sprouted vigorously from above-ground stems after fire occurring at some point in the previous 2–3 years. At the time of the type collection, plants were robust and vigorous in areas that had been burned and only represented by a few weak ramets in unburned areas dominated by *Triodiapungens* tussocks.

Pollination biology of the species is unknown, but, like other Australian congeners, the flowers are likely buzz pollinated by bees in the genera *Xylocopa* and *Amegilla* (Apidae; see Anderson and Symon 1988; Switzer et al. 2016) and likely to present high levels of pollen nutritional reward – although with slightly differential rewards available to pollen foragers from male versus functionally female flowers (Ndem-Galbert et al. 2021). A small set (*N* = 10) of ex situ hand pollinations conducted for this study showed that inaperturate pollen produced by functionally female flowers does not lead to fruit set when used to pollinate other females. This suggests that reproduction in *S.scalarium* is dependent on intersexual male-to-female outcrossing via biotic pollination like in other dioecious *Solanum* species.

Seed dispersal mechanism for this species is also unknown, although young fleshy fruits are mostly enclosed in a spiny calyx that gradually reflexes to some degree as fruits become dry and bony (Fig. 6D, E), suggesting that endozoochory is less likely than either ectozoochory (as a trample burr) or passive dispersal (see Symon 1979b; Martine et al. 2019). Peoples of the Walmajarri language area of the Kimberley region (west of this distribution) report that the fruits of *S.dioicum* (*kara*) are eaten by *Osphranterrufus* (Desmarest, 1822) (plains or red kangaroos; Doonday et al. 2013), and CTM has seen bustard birds (*Ardeotisaustralis* (Gray, 1829)) picking apart *S.dioicum* fruits near the northwest Kimberley coast (Martine et al. 2019). However, there is no published evidence that any extant animal acts as an effective seed dispersal agent of taxa within the “Kimberley dioecious clade” (see Martine et al. 2016b). Notably, seeds removed from green fruits (enclosed in calyces) of the type collection as well as seeds removed from older brown fruits (calyces reflexed) in cultivation were both germinable, suggesting that effective seed dispersal might happen at either stage.

**Figure 5.**
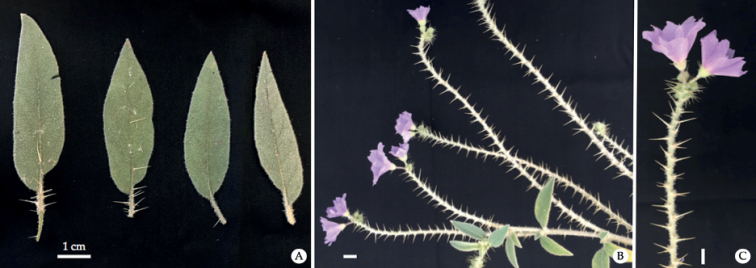
Functionally male individuals of *Solanumscalarium* in cultivation: **A** leaf shape and **B, C** inflorescence axes of male individuals; note the distinctive spreading straight prickles that give the inflorescence axis a ladder-like appearance. (Photos by T.M. Williams.).

**Figure 6.**
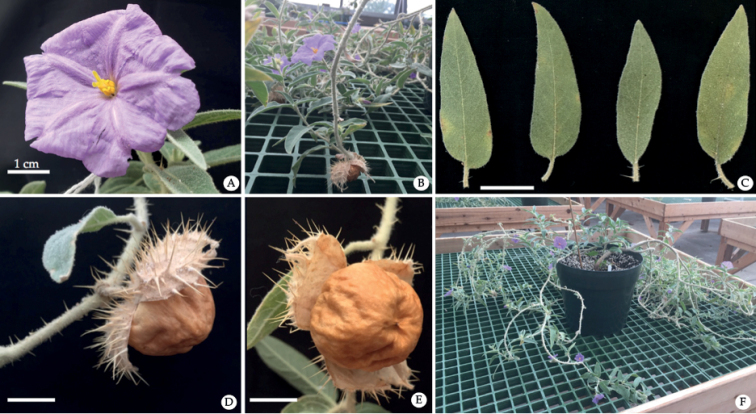
Functionally female individuals of *Solanumscalarium* in cultivation: **A** flower **B** reproductive branch **C** leaf shape **D, E** reflexing of calyx around brown, bony fruits and **F** overall habit. (Photos by T.M. Williams.).

**Figure 7.**
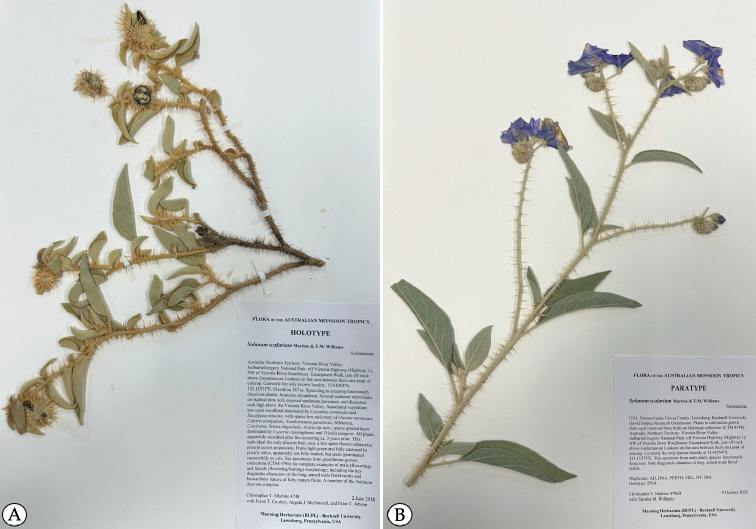
**A** Holotype of *Solanumscalarium* (C.T. Martine 4748; female fruiting specimen) **B** paratype of *S.scalarium* (C.T. Martine 4796B; male specimen). (Photos by M. Sain.).

#### Phenology.

Plants encountered on 2 June 2018 were largely sterile, with several withered flowers and two mature fruits. The low fruit set in the population at the time of collection suggests that *S.scalarium* typically flowers earlier in the calendar year.

#### Etymology.

Latin *scalare* from *scala*, ladder or stair, and suffix *aris*, pertaining to; the epithet *scalarium* is the genitive plural of *scalare*, indicating ladder-like appearance of staminate inflorescence rachises, which are conspicuously and unusually armed with relatively stout, spreading, straight prickles resembling ladder steps. It is also a nod to the type locality at the Escarpment Walk, Judbarra/Gregory National Park, where stone stairs lead from the car park up to the habitat of this species. By choosing this name we acknowledge the access these steps provide to the newly-described species as well as the importance of providing broad access to nature, outdoor recreation, and scientific discovery. We suggest the use of Garrarnawun Bush Tomato for the English-language common name of the species in recognition of the Garrarnawun Lookout near where the type collection was made, a traditional meeting place of the Wardaman and Nungali-Ngaliwurru peoples whose lands overlap in this area (Parks and Wildlife Commission of the Northern Territory 2021).

#### Preliminary conservation status.

While we expect that more localities for *S.scalarium* are likely to be found given the prevalence of similar (and less accessible) outcrops in the immediate region of the type collection, at present it is known from one protected (though frequently-visited) collection site in Judbarra/Gregory National Park (Fig. 4). Based on IUCN Red List Categories (IUCN 2012), *S.scalarium* should be considered Data Deficient (DD).

#### Specimens examined.

United States. Pennsylvania: Cultivated in Bucknell University: (Lewisburg) Burpee Research Greenhouse (staminate flowers/inflorescences, functionally female flowers, and fruits), 9 Oct 2020*CT Martine & TM Williams 4796.* (To be distributed to AD, BM, BUPL [Fig. 7b], DNA, NY, PERTH, US).

#### Diagnostic couplets.

A comprehensive “Kimberley dioecious clade” key, including newly-recognized species, is forthcoming (Barrett and Barrett in prep). The most complete key to date can be found in Barrett (2013), which lumps the numerous variations of *S.dioicum* sensu lato as a single taxon. The following couplets may be inserted where *S.dioicum* occurs at couplet 60 in the key in Barrett (2013) and supplants the single replacement couplet 60a [previously published in Martine et al. (2016c)].

[Barrett 2013; couplet 60]

**Table:** 

60a	Plants less than 1 m tall, many-branched; stems moderately to densely prickly; leaf indumentum silvery/rusty/yellow, overall aspect silvery-green, yellow-green, or reddish-green; stigma deeply bifid, the lobes 2–5 mm long; calyx not fully enclosing mature fruit	**60b**
60a	Plants more than 1 m tall, few-branched and conspicuously “Y”-shaped in form; stems very prickly; leaf indumentum silvery, overall aspect silvery-blue; stigma shallowly bifid, the lobes 0.5–1 mm long; calyx fully enclosing the mature fruit	***Solanumossicruentum* Martine & J.Cantley**
60b	Plants many-branched; stems moderately prickly; leaf indumentum silvery or rusty, overall aspect silvery-green, yellowish green, or reddish green; stigma lobes 2–5 mm long; mature fruits green and fleshy; male floral rachis typically unarmed	***Solanumdioicum* W.Fitzg.**
60b	Plants many-branched and spreading decumbent in form; stem densely prickly; leaf indumentum yellow, overall aspect yellow-green; stigma lobes 1.5–2 mm; mature fruits light green to yellow-orange and fleshy, becoming tan and bony hard; male floral rachis armed	***Solanumscalarium* Martine & T.M.Williams**

## ﻿Discussion

*Solanumscalarium* is the latest in a series of newly-described functionally dioecious species from the “Kimberley dioecious clade” (see Martine et al. 2011, 2013, 2016c; Barrett 2013) a group that is still rife with undescribed species lumped under the umbrella of *S.dioicum* (Barrett 2013). Although some of the variation within the clade is subtle and/or continuous, *S.scalarium* can be distinguished from all known members by the combination of spreading decumbent habit and male inflorescence rachis armed with relatively stout, spreading, straight prickles. Complex habitat and environmental characteristics, coupled with climate fluctuations over the last two million years, have been drivers of the high species diversity and speciation events throughout Australia (Bowman et al. 2010; Edwards et al. 2017; Edwards et al. 2018). There are particularly high levels of plant diversity and newly described species within the AMT (Bowman et al. 2010; Barrett 2013; Edwards et al. 2017; Edwards et al. 2018; Martine et al. 2019).

Forthcoming phylogenomic work (e.g., McDonnell and Martine in prep) should aid in resolving what has been a decades-long effort to gradually assign species names to recognizable local forms in this complex group. In the meantime, the best course of action continues to be collecting all forms of “*Solanumdioicum*” when they are encountered such that the variation within the group continues to be captured in herbarium collections (see Heberling et al. 2019; Thiers 2020).

The scientific name and English-language common name proposed here acknowledge the critical importance of maintaining equitable and safe access to outdoor spaces, the Garrarnawun Lookout being a poignant example of shared use of special places.

## Supplementary Material

XML Treatment for Solanum scalarium

## References

[B1] Anderson GJ Symon DE (1988) Insect foragers on *Solanum* flowers in Australia. Annals of the Missouri Botanical Garden 75(3): 842–852. 10.2307/2399372

[B2] Australian Government Department of the Environment and Energy (2015) Judbarra/Gregory National Park, Northern Territory 2015: A Bush Blitz Survey Report. https://bushblitz.org.au/wp-content/uploads/2017/05/Judbarra-Report.pdf [accessed 2.02.2022]

[B3] Barrett RL (2013) *Solanumzoeae* (Solanaceae), a new species of bush tomato from the North Kimberley, Western Australia. Nuytsia 23: 5–21.

[B4] Bean AR (2004) The taxonomy and ecology of Solanumsubg.Leptostemonum (Dunal) Bitter (Solanaceae) in Queensland and far north-eastern New South Wales, Australia. Austrobaileya 6: 639–816.

[B5] Bean AR Albrecht DE (2008) *Solanumsuccosum* A.R.Bean & Albr. (Solanaceae), a new species allied to *S.chippendalei* Symon. Austrobaileya 7: 669–675.

[B6] Bean AR (2016) Two new species of *Solanum* (Solanaceae) from the Northern Territory, Australia. Austrobaileya 9: 524–533.

[B7] Bowman DMJS Brown GK Braby MF Brown JR Cook LG Crisp MD Ford F Haberle S Hughes J Isagi Y Joseph L McBride J Nelson G Ladiges PY (2010) Biogeography of the Australian monsoon tropics. Journal of Biogeography 37(2): 201–216. 10.1111/j.1365-2699.2009.02210.x

[B8] Brennan K Martine CT Symon DE (2006) *Solanumsejunctum* (Solanaceae), a new functionally dioecious species from Kakadu National Park, Northern Territory, Australia. The Beagle. Records of the Museums and Art Galleries of the Northern Territory 22: 1–7.

[B9] Cowie IC Cuff NJ Lewis DL Jobson PC [Eds] (2017) Checklist of the vascular plants of the Northern Territory. Northern Territory Herbarium, Department of Environment and Natural Resources, Palmerston, Northern Territory.

[B10] Craven LA (1998) A result of the 1996 Mueller Commemorative Expedition to northwestern Australia: *Melaleucatriumphialis* sp. nov. (Myrtaceae). Muelleria 11: 1–4. 10.5962/p.198402

[B11] Doonday B Samuels C Clancy EM Milner J Chungulla R Whisputt M Yoomarie S Lulu V Johns A Brown S Vernes T Richards E Wightman G (2013) Walmajarri Plants and Animals: Aboriginal Knowledge from the Paruku Indigenous Protected Area, Southern Kimberley. Northern Territory Botanical Bulletin No 42 Department of Land Resource Management, NTG, Broome.

[B12] Edwards RD Crisp MD Cook DH Cook LG (2017) Congruent biogeographical disjunctions at a continent-wide scale: Quantifying and clarifying the role of biogeographic barriers in the Australian tropics. PLoS ONE 12(4): e0174812. 10.1371/journal.pone.0174812 PMC5380322 28376094

[B13] Edwards RD Crisp MD Cook LG (2018) Species limits and cryptic biogeographic structure in a widespread complex of Australian monsoon tropics trees (broad-leaf paperbarks: *Melaleuca*, Myrtaceae). Australian Systematic Botany 31: 495–503. 10.1071/SB18032

[B14] Gagnon E Hilgenhof R Orejuela A McDonnell AJ Sablok G Aubriot X Giacomin L Gouvêa Y Bohs L Dodsworth S Martine CT Poczai P Knapp S Särkinen T (2022) Phylogenomic data reveal hard polytomies across the backbone of the large genus *Solanum* (Solanaceae). American Journal of Botany 109: 1–22. 10.1002/ajb2.1827 35170754 PMC9321964

[B15] Hayes D Jordon-Thaden IE Cantley JT McDonnell AJ Martine CT (2019) Integrated pest management in the academic small greenhouse setting: A case study using *Solanum* spp. (Solanaceae). Applications in Plant Sciences 7(8): e11281. 10.1002/aps3.11281 PMC6711345 31467804

[B16] Heberling JM Prather LA Tonsor SJ (2019) The changing uses of herbarium data in an era of global change: An overview using automated content analysis. Bioscience 69(10): 812–822. 10.1093/biosci/biz094

[B17] Hilgenhof R Gagnon E Knapp S Aubriot X Tepe EJ Bohs L Giacomin LL Gouvêa Y Stehmann JR Martine CT Orejuela A Orozco CI Peralta IE Särkinen T (In review) Morphological trait evolution in *Solanum* (Solanaceae): evolutionary lability of key taxonomic characters.

[B18] IUCN (2012) IUCN Red List Categories and Criteria: Version 3.1. 2^nd^ Edn.

[B19] Jobson PC (2014) Two rare new species of *Isotropis* (Fabaceae: Faboideae: Mirbelieae) from tropical northern Australia. Telopea 17: 347–354. 10.7751/telopea20148179

[B20] Knapp S (2013) A revision of the Dulcamaroid Clade of *Solanum* L. (Solanaceae). PhytoKeys 22(0): 1–432. 10.3897/phytokeys.22.4041 PMC3689140 23794937

[B21] Lacey LM Cantley JT Martine CT (2017) *Solanumjobsonii*, a novel andromonoecious bush tomato species from a new Australian national park. PhytoKeys 82: 1–13. 10.3897/phytokeys.82.12106 PMC5543266 28794678

[B22] Martine CT Vanderpool D Anderson GJ Les DH (2006) Phylogenetic relationships of andromonoecious and dioecious Australian species of SolanumsubgenusLeptostemonumsectionMelongena: Inferences from ITS sequence data. Systematic Botany 31(2): 410–420. 10.1600/036364406777585801

[B23] Martine CT Anderson GJ Les DH (2009) Gender-bending aubergines: Molecular phylogenetics of cryptically dioecious *Solanum* in Australia. Australian Systematic Botany 22(2): 107–120. 10.1071/SB07039

[B24] Martine CT Lavoie EM Tippery NP Vogt FD Les DH (2011) DNA analysis identifies *Solanum* from Litchfield National Park as a lineage of *S.dioicum*. Northern Territory Naturalist 23: 29–38. 10.5962/p.295491

[B25] Martine CT Symon DE Capaldi Evans E (2013) A new cryptically dioecious species of bush tomato (*Solanum*) from the Northern Territory, Australia. PhytoKeys 30: 23–31. 10.3897/phytokeys.30.6003 PMC3881354 24399898

[B26] Martine CT Frawley ES Cantley JT Jordon-Thaden IE (2016a) *Solanumwatneyi*, a new bush tomato species from the Northern Territory, Australia named for Mark Watney of the book and film “The Martian”. PhytoKeys 61: 1–13. 10.3897/phytokeys.61.6995 PMC4816977 27081345

[B27] Martine CT Boni AJ Capaldi EA Lionheart G Jordon-Thaden IE (2016b) Evidence of rock-dwelling macropod seed dispersal in a tropical monsoon community. Northern Territory Naturalist 27: 68–77. 10.5962/p.295470

[B28] Martine CT Cantley JT Frawley E Butler A Jordon-Thaden IE (2016c) New functionally dioecious bush tomato, *Solanumossicruentum*, may utilize “trample burr” seed dispersal. PhytoKeys 63: 19–29. 10.3897/phytokeys.63.7743 PMC4956925 27489475

[B29] Martine CT Jordon-Thaden IE McDonnell AJ Cantley JT Hayes DS Roche MD Frawley ES Gilman IS Tank DC (2019) Phylogeny of the Australian *Solanumdioicum* group using seven nuclear genes, with consideration of Symon’s fruit and seed dispersal hypotheses. PLoS ONE 14(4): e0207564. 10.1371/journal.pone.0207564 PMC6472733 30998778

[B30] McDonnell AJ Martine CT (2020) Phylogenomics and breeding system evolution of Australian *Solanum* (“*S.dioicum* group”, Solanaceae). Botany 2020: Virtual Annual Meeting of the Botanical Society of America. [online abstract:] http://2020.botanyconference.org/engine/search/index.php?func=detail&aid=238

[B31] McDonnell AJ Wetreich HB Cantley JT Jobson P Martine CT (2019) *Solanumplastisexum*, an enigmatic new bush tomato from the Australian Monsoon Tropics exhibiting breeding system fluidity. PhytoKeys 124: 39–55. 10.3897/phytokeys.124.33526 31258372 PMC6592974

[B32] Ndem-Galbert JR Hall J McDonnell AJ Martine CT (2021) Differential reward in “male” versus “female” pollen of functionally dioecious *Solanum* (Solanaceae). American Journal of Botany 108(11): 2282–2293. 10.1002/ajb2.1765 34643272 PMC9298796

[B33] Parks and Wildlife Commission of the Northern Territory (2021) Judbarra/Gregory National Park Fact Sheet. Katherine NT 0850. https://nt.gov.au/__data/assets/pdf_file/0009/278442/judbarra-gregory-national-park-fact-sheet-and-map.pdf [accessed 2.02.2022]

[B34] Purdie RW Symon DE Haegi L (1982) Flora of Australia. Solanaceae. Vol. 29. Australian Government Publishing Service, 1–227.

[B35] Särkinen T Olmstead RG Bohs L Knapp S (2013) A phylogenetic framework for evolutionary study of the nightshades (Solanaceae): A dated 1000-tip tree. BMC Evolutionary Biology 13(1): 214. 10.1186/1471-2148-13-214 24283922 PMC3850475

[B36] Switzer CM Hogendoorn K Ravi S Combes SA (2016) Shakers and head bangers: Differences in sonication behavior between Australian *Amegillamurrayensis* (blue-banded bees) and North American *Bombusimpatiens* (bumblebees). Arthropod-Plant Interactions 10(1): 1–8. 10.1007/s11829-015-9407-7

[B37] Symon DE (1979b) Fruit diversity and dispersal in *Solanum* in Australia. Journal of the Adelaide Botanic Gardens 1: 321–331.

[B38] Symon DE (1981) A revision of the genus *Solanum* in Australia. Journal of the Adelaide Botanic Gardens 4: 1–367.

[B39] Thiers BM (2020) Herbarium: The Quest to Preserve and Classify the World’s Plants. Journal of the Botanical Research Institute of Texas 15: 524.

[B40] Vorontsova MS Stern S Bohs L Knapp S (2013) African spiny Solanum (subgenus Leptostemonum, Solanaceae): A thorny phylogenetic tangle. Botanical Journal of the Linnean Society 173(2): 176–193. 10.1111/boj.12053

[B41] Walsh NG Albrecht DE (1998) A new species of *Eucalyptus* (series *Subexsertae*) from the Northern Territory. Muelleria 11: 41–44. 10.5962/p.198406

[B42] Whalen MD (1984) Conspectus of species groups in SolanumsubgenusLeptostemonum. Gentes Herbarum 12: 179–282.

